# Heparin Anticoagulant for Human Bone Marrow Does Not Influence In Vitro Performance of Human Mesenchymal Stromal Cells

**DOI:** 10.3390/cells9071580

**Published:** 2020-06-29

**Authors:** Yvonne Roger, Laura Burmeister, Anika Hamm, Kirsten Elger, Oliver Dittrich-Breiholz, Thilo Flörkemeier, Andrea Hoffmann

**Affiliations:** 1 Department of Orthopedic Surgery, Graded Implants and Regenerative Strategies, OE 8893, Hannover Medical School (MHH), 30625 Hannover, Germany; Roger.Yvonne@mh-hannover.de (Y.R.); Laura.Burmeister@gmx.net (L.B.); Hamm.Anika@mh-hannover.de (A.H.); Elger.Kirsten@mh-hannonver.de (K.E.); 2 Lower Saxony Centre for Biomedical Engineering, Implant Research and Development (NIFE), 30625 Hannover, Germany; 3 Research Core Unit Genomics, OE 9415, Hannover Medical School (MHH), 30625 Hannover, Germany; Dittrich.Oliver@mh-hannover.de; 4 Department of Orthopedic Surgery (Annastift), OE 6270, Hannover Medical School (MHH), 30625 Hannover, Germany; Thilo.Floerkemeier@g-o-hannover.de

**Keywords:** unfractionated heparin, ASA status, density gradient, cell heterogeneity, osteogenic differentiation, Alizarin Red S stain, marker genes, transcriptome analyses

## Abstract

Mesenchymal stromal cells (MSCs) are a promising cell source for tissue engineering and regenerative medicine. In our lab, we found that MSC preparations from bone marrow of many different donors had a limited capacity of in vitro differentiation into osteogenic and chondrogenic lineages—a capacity claimed to be inherent to MSCs. The current study was designed to test the hypothesis that the amount of heparin used as anticoagulant during bone marrow harvest had an inhibitory influence on the in vitro differentiation capacity of isolated MSCs. Bone marrow was obtained from the femoral cavity of twelve donors during total hip arthroplasty in the absence or presence of heparin. No coagulation was observed in the absence of heparin. The number of mononuclear cells was independent of heparin addition. Isolated MSCs were characterized by morphology, population doubling times, expression of cell surface antigens and in vitro differentiation. Results of these analyses were independent of the amount of heparin. Transcriptome analyses of cells from three randomly chosen donors and quantitative realtime PCR (qRT-PCR) analysis from cells of all donors demonstrated no clear effect of heparin on the transcriptome of the cells. This excludes heparin as a potential source of disparate results.

## 1. Introduction

Mesenchymal stromal cells (MSCs) constitute a major cell population which is used in research, translational studies, and clinical applications, and bone marrow is one major tissue source. MSCs are largely isolated and described by minimal criteria which were defined by the International Society for Cell Therapy in 2006: Fibroblastic cells with plastic-adherent growth in cell culture, with the presence and absence of selected cell surface antigens and with the capacity to differentiate into osteoblasts, adipocytes, and chondrocytes in vitro [1,2]. Unfortunately, cell culture conditions and methods for examination of these criteria vary largely between individual labs. In research studies bone marrow is frequently obtained from patients during diagnostic or therapeutic intervention. The health state of these donors might be unclear. Age, gender, and other parameters such as inflammatory status have an important but largely uncontrollable impact and consequently calls for action to obtain more information. Furthermore it is known that culture conditions including the choice of fetal calf serum and use of different proteases during passaging can have an immense effect on the performance of the cells. To start at the very beginning with tissue harvest for MSC isolation, it might be important to know how the bone marrow is harvested and treated before extraction with respect to anti-coagulation—next to additional interesting aspects like the site (iliac crest or long bones) and mode of bone marrow harvest (aspiration or retrieval from an opened bone).

In the published literature, many publications do not give any information on whether their bone marrow was subjected to anticoagulant treatment in order to prevent clotting. Secondly, if so, the chemical nature of the used anticoagulant is often not indicated. Different anticoagulants are in current use for blood and bone marrow: Acid citrate dextrose, citrate phosphate dextrose, ethylenediamine tetraacetic acid, heparin (Li^+^-, Na^+^-, K^+^-variants), and others. Thirdly, the applied dosages vary largely. Some studies indicate that mere rinsing of syringes with heparin solution was applied [3] but others used 100 IU/mL bone marrow [4]. Two types of heparins are widely used, high molecular weight unfractionated heparin and low molecular weight fractionated heparin. In general, there is not only a difference in molecular weight, it is also shown that there is a more unpredictable effect for unfractionated heparin [5]. Chemically, heparin constitutes a glycosaminoglycan. It acts primarily through a complex with antithrombin III thereby preventing formation of fibrin from fibrinogen and consequently prevents blood clotting. In addition, with a comparably high degree of sulfation and consequently strong negative charge, heparin belongs to a promising class of bio-active substances that can potentially enhance the biological effects of proteins due to binding and sustained and controlled release of cytokines including growth factors (reviewed recently by [6]).

The issue of anticoagulation may become particularly relevant when systemic administration of MSCs is planned. Therefore, several studies have addressed the effect of heparin on performance of bone marrow MSCs (BM-MSCs). BM-MSCs from different species were applied intravenously in animal models in which heparin co-application successfully prevented cell coagulation in vivo [7]. Human BM-MSCs injected into infarcted rabbit hearts significantly reduced the fibrotic area when combined with subcutaneous application of heparin [8]. Non-modified and modified heparin was also used as an appealing biomaterial in many studies in combination with MSCs and other cell types, attractive for local use in vitro and in vivo [9,10]. Here, heparin seemed to facilitate and increase the proliferation of MSCs.

Studies have also been performed with constant heparin addition during BM-MSC culture in vitro [11,12]. Promotion of osteogenic and reduction of adipogenic differentiation were found upon use of 20 U/mL unfractionated heparin [11]. Contrasting with these results, another study showed that osteogenic, chondrogenic, and adipogenic differentiation were scarcely affected by long-term culture with 160 ng/mL heparin [12]. The expression of the surface antigens CD49a, CD73, and CD105 did not show notable changes after passaging in the presence of 160 ng/mL heparin [12]. Dependent on the concentration of heparin and the MSC passage, different effects on proliferation were observed [12]. The authors concluded that heparin should not be used as culture supplement for expansion and maintenance of naïve human MSCs [10].

Effects of heparin addition to bone marrow preparations and the ensuing consequences for isolation and functionality of BM-MSCs have not yet been investigated systematically. One study showed that heparin blocked SDF1/CXCR4-signaling in mononuclear cells from bone marrow (without expansion in adherent cell culture), thereby interfering with homing and migration of the cells [13]. However, culture-expanded MSCs derived from these mononuclear cells were not investigated in the study [13]. In another analysis, human BM-MSCs as well as stromal cells from umbilical cord and white adipose tissue were isolated and propagated in human platelet lysate-supplemented media with or without heparin [14]. Here, transcriptome analyses revealed regulation of distinct gene sets by heparin including signaling cascades involved in proliferation, cell adhesion, apoptosis, inflammation, and angiogenesis, depending on the stromal cell origin [14]. Despite these observations heparin had no substantial effect on long-term proliferation and in vitro tri-lineage differentiation of stromal cells [14].

Considering this state of knowledge, we were interested in obtaining more detailed information on the influence of heparin on human bone marrow during the initial step for subsequent MSC isolation—the tissue sampling process itself—and the quality of the subsequently isolated MSCs. This was reinforced by our finding that, despite the use of standard protocols for cell isolation and differentiation assays, the large majority of our cell preparations obtained from different human orthopedic bone marrow donors did not conform well with the International Society for Cell Therapy minimal criteria, in particular with respect to their in vitro differentiation potential into osteogenic or chondrogenic lineages. Colleagues from our university reported similar findings for bone marrow samples from healthy volunteers (unpublished results). Therefore, the present study was designed with twelve bone marrow donations obtained during implantation of a total hip arthroplasty. Three aliquots of bone marrow from the same donor, 5 mL each, were either not anti-coagulated or supplemented with two different concentrations of heparin. Mononuclear cells including MSCs were subsequently isolated by density gradient centrifugation within four hours of bone marrow harvest. Plastic-adherent cells were expanded and characterized for the number of mononuclear cells obtained after density gradient centrifugation, morphology, proliferation, expression of cell surface antigens, in vitro differentiation, and gene expressions.

## 2. Materials and Methods

### 2.1. Materials

Heparin was supplied as sodium salt, from pig intestine mucosa, unfractionated (UFH), distributor Rotexmedica (25.000 IU/ 5 mL).

### 2.2. Ethics Statement

Ethical approval was obtained from the ethical committee of Hannover Medical School (#565-2009, 28.08.2009, #565-2016, 22.04.2016). Samples were collected in accordance with the Declaration of Helsinki of 1975, revised in 2013, after written informed consent of all donors. All personal information except age and gender was deleted.

### 2.3. Bone Marrow Harvest

No preconditioning of the patients was performed before surgery. In particular systemic anticoagulation was not used. Donor information is presented in Table 1 and Supplemental Table S1. During preparation of the femoral cavity for the implantation of the femoral stem for a total hip arthroplasty, the femoral canal was opened, bone marrow was pressed out and collected intraoperatively as described [15]. During this process, no coagulation was observed. For each donor, three aliquots of 5 mL bone marrow were collected and each sample was prepared as follows: Condition 1 with no heparin in the syringe, condition 2 supplemented with 1.5 mL heparin (i.e., 1,500 IU/mL bone marrow), and condition 3 supplemented with 3.5 mL heparin (i.e., 3500 IU/mL bone marrow). The quality of bone marrow was assessed intraoperatively macroscopically and graded from 1 (excellent) to 6 (inferior) (Table 2). “Excellent” means that the bone marrow fluid contained a high amount of bone marrow cells and only few blood cells, i.e., was not reddish but yellow turbid due to the presence of fat cells. “Inferior” designates a small percentage of bone marrow cells and a high percentage of blood. In addition, the sequence of sample preparations was varied intraoperatively i.e., the sample without heparin was not always the first sample to be prepared (Table 2) in order to avoid introducing bias. The bone marrow samples were processed in the laboratory for isolation of mononuclear cells by density gradient centrifugation as quickly as possible, usually within 4 h. Only bone marrow from donor J was stored overnight. For processing of identical volumes, 0.9% sodium chloride (B. Braun, Melsungen, Germany) were used to make up a final sample volume of 8.5 mL: the sample with no heparin was diluted with 3.5 mL 0.9% NaCl, the sample with 1.5 mL heparin was diluted with 2 mL 0.9% NaCl and the sample with 3.5 mL heparin was not diluted. Sodium chloride was chosen as it has similarities to the commercial heparin preparation which contained benzyl alcohol, sodium chloride, and water for injection (exact composition proprietary knowledge of the manufacturer).

### 2.4. Isolation and Culture of Human MSCs from Bone Marrow

The bone marrow preparations were further diluted fourfold with phosphate-buffered saline without Ca^2+^, Mg^2+^ (PBS). The samples were filtrated (cell strainer, 100 µm) to remove bone debris and then were slowly applied to the density gradient material using a ratio of 1 volume Biocoll (Biochrom, Berlin, Germany, L6115; ρ = 1.077 g/mL) to 2 volumes of diluted bone marrow in conical 50 mL centrifuge tubes. After centrifugation at 500× *g* for 30 min at room temperature without brakes, the mononuclear cells directly above the density gradient material were recovered, washed once with PBS, pelleted and suspended in 10 mL medium. Cell counting was performed manually with a Fuchs-Rosenthal chamber with the cell suspensions diluted 1:10. The retrieved mononuclear cells were cultured in vessels for growth of adherent cells at a plating density of 500,000 mononuclear cells/cm^2^. The MSC expansion medium was Dulbecco’s Modified Eagle´s Medium (DMEM) Low Glucose (1 g/L glucose, Biochrom, FG0415) supplemented with 10% (*v*/*v*) fetal bovine serum (not heat-inactivated, Thermo Fisher Scientific, Schwerte, Germany, “HyClone”, SV30160.03), 25 mM 4-(2-hydroxyethyl)-1-piperazineethanesulfonic acid (HEPES, Biochrom), 1% (100 U/mL/100 µg/mL) penicillin/streptomycin (Biochrom) and 2 ng/mL human recombinant fibroblast growth factor-2 (FGF-2, from *Escherichia coli*, PeproTech, Hamburg, Germany). The cells were cultured at 37 °C with 5% CO_2_ at 85% humidity. After 24 h the medium was replaced by fresh MSC expansion medium and subsequent media changes were performed every 3–4 days. The cells were passaged at a confluence of around 70% by using 0.05%/0.02% (*w*/*v*) trypsin-ethylenediamine tetraacetic acid solution (Trypsin/EDTA; Biochrom L2153) and passaged with 2000 cells per cm² for a maximum of five passages. Morphology was documented by phase contrast microscopy. Proliferation was documented by manual cell-counting with a Fuchs-Rosenthal chamber. Population doubling times (PDT, in hours) were calculated between passages 1 and 2 using the formula:

PDT = (time (between p. 2 and p. 1 in hours) * ln)/ln (total cells in p. 2/total cells in p. 1).(1)

Detailed information on isolation and propagation of the different MSC preparations is compiled in Table 2. Bone marrow from twelve donors (A to L) was compared in the present study. In addition, preliminary experiments were conducted with cell samples from donors AA—SS which had been obtained previously by our regular procedure of mixing 2.5 mL heparin with 10 mL bone marrow (1.250 IU/mL bone marrow).

### 2.5. Flow Cytometric Measurements (FCM)

One T25 flask was used per condition and donor after the cells had reached a confluence of about 80% in passages 2 to 4. After washing with PBS, the cells were detached with Trypsin/EDTA and portioned into 5 mL round-bottom test tubes (Corning, Bedford, USA). Blocking was not required due to a specifically mutated human IgG1 Fc region which eliminates their binding to Fc receptors. Samples were subsequently stained with antibodies against CD14, CD19, CD45, CD73, CD90, CD105, HLA-DR, and the respective isotype-matching IgG control antibodies diluted in PBS with 2% (*w*/*v*) bovine serum albumin (BSA, 100 µl/ sample) at 4 °C for 15 min. The following monoclonal anti-human antibodies were used: CD14-APC (allophycocyanin, clone REA599, IgG1; Miltenyi Biotech, Bergisch-Gladbach, Germany), CD19-Viogreen (clone REA675, IgG1; Miltenyi Biotech), CD45-APC-Vio770 (clone REA747, IgG1; Miltenyi Biotech), CD73-PEVio615 (clone REA804, IgG1; Miltenyi Biotech), CD90-FITC (fluorescein isothiocyanate; clone REA897, IgG1; Miltenyi Biotech), CD105-Vioblue (clone REA794, IgG1; Miltenyi Biotech), HLA-DR-PE (human leukocyte antigen-DR with phycoerythrin; clone REA805, IgG1; Miltenyi Biotech). Afterwards, cells were washed twice with PBS/BSA and analyzed by flow cytometry using MACSQuant10 (Miltenyi Biotech) and FCSalyzer 0.9.14-alpha software. The entire flow cytometric procedure was performed by one person for all samples to minimize individual variance with respect to gating.

### 2.6. In Vitro Differentiation of MSCs

Adipogenic differentiation was induced by plating 5000 cells/cm^2^ in sixwell-plates (Greiner, Frickenhausen, Germany). Upon reaching confluence, differentiation was induced by using DMEM with high glucose content (4.5 g/L glucose, Biochrom, Berlin, Germany, FG0435) with 10% (*v*/*v*) fetal bovine serum, 20 mM HEPES, 1% (100 U/mL/100 µg/mL) penicillin/streptomycin (Biochrom) and supplemented with 1 µM dexamethasone, 60 µM indomethacine (Sigma, Munich, Germany), 500 µM 3-isobutyl-1-methylxanthine (Sigma), and 10 µg/mL insulin (from bovine pancreas, Sigma I0516). Differentiation was performed for a maximum of 14 days and medium was replaced twice a week. RNA was isolated from all samples as described below.

Osteogenic differentiation was induced by plating 5000 cells/cm^2^ in sixwell-plates (Greiner) coated with 2% (*w*/*v*) gelatin B (dissolved in water; Sigma G6650 from bovine skin, autoclaved). Upon reaching confluence (termed day 0 of differentiation), differentiation was induced by supplementation of basal medium consisting of DMEM with Low Glucose with 10% (*v*/*v*) fetal bovine serum, 20 mM HEPES, 1% (100 U/mL / 100 µg/mL) penicillin/streptomycin (Biochrom) with either 100 nM dexamethasone (Sigma) or 200 ng/mL recombinant human bone morphogenetic protein-2 (BMP-2, from *Escherichia coli,* kind gift from G. Gross, Helmholtz Centre for Infection Research, Braunschweig [16]) as indicated in the results, plus 50 µM ascorbate-2-phosphate (Sigma) and 10 mM beta-glycerophosphate (Sigma) in both induction protocols. Differentiation was performed for 27 days. Medium was replaced twice a week. MSCs from donor G started to detach at day 19 of differentiation. Therefore, this donor was not included in the analyses. Available cell numbers from donor L were too low so that no osteogenic differentiation experiment was started. RNA was isolated from all samples as described below. Parallel cultures were fixed for cytochemical staining or for determination of the calcium-to-phosphate ratio in the cell layer as described below.

Chondrogenic differentiation was induced in a three-dimensional pellet culture. The required number of cells (for each pellet 1.25 × 10^5^ cells) was transferred into a 15 mL-tube (Greiner) and centrifuged for 5 min at 200× *g*. The supernatant was removed and the pellet washed with DMEM with high glucose content (Biochrom, FG0435) with 20 mM HEPES, 1% (100 U/mL / 100 µg/mL) penicillin/streptomycin (Biochrom). After another centrifugation step (5 min at 200× *g*), the medium was discarded and the generated pellet was mixed with induction medium made of DMEM with high glucose content (Biochrom, FG0435) with 20 mM HEPES, 1% (100 U/mL / 100 µg/mL) penicillin/streptomycin (Biochrom), 100 nM dexamethasone, 1x ITS^+^ (Sigma I3146), 170 µM ascorbate-2-phosphate, 1 mM sodium pyruvate (Biochrom), 350 µM proline (Carl Roth) and 10 ng/mL human recombinant TGF-β_3_ (*Escherichia coli*, PeproTech, Hamburg, Germany). The cell suspension was portioned into 96-well plate with round bottoms (polypropylene, Corning #3879 via Sigma-Aldrich) with 200 µL per well corresponding to 1.25 × 10^5^ cells per well. Control pellets were cultured without TGF-β_3_. Medium was changed every second day. After 28 days, RNA was isolated from the cell pellets as described below. Three pellets were pooled for RNA isolation which was performed in duplicates (six pellets in total). One separate pellet was used for analysis of the glycosaminoglycan-to-DNA ratio.

For donor J, cells isolated with the addition of 3.5 mL heparin did not proliferate substantially after passage 2 (see Table 2). Therefore, the population doubling time could not be calculated and the cells could not be subjected to osteo- or chondrogenic differentiation (marked by x in Figures 2, 3, 5, 6 and 7).

### 2.7. RNA Isolation and cDNA Synthesis

Duplicate samples from differentiated cells (osteogenic and adipogenic induction) were lysed in 350 µL buffer RLT (Qiagen, Hilden, Germany), lysates were stored at −80 °C. RNA was subsequently isolated with the RNeasy kit (Qiagen #74106) according to the instructions of the manufacturer including on-column-digestion with DNase I. Synthesis of cDNA was performed from 1 µg of RNA with oligo-dT_18_-primers according to the manufacturer´s suggestions (Fermentas, Bremen, Germany). Total RNA from chondrogenic pellets was isolated with a homogenizer from Bertin technologies (PreCellys 24 lysis and homogenization; 2 × 30 s, 6000 rpm with 40 s pause) and a Precellys^®^ RNA Kit (732-3122, VWR, Langenfeld, Germany). The isolation was carried out according to the manufacturer’s manual. Synthesis of cDNA was performed with 200 ng of RNA including treatment with DNase I and oligo-dT_18_-primers according to the manufacturer´s suggestions (Fermentas).

### 2.8. Histological Staining for Osteogenic and Adipogenic Differentiation

Cells were fixed with 4% (*w*/*v*) paraformaldehyde (Carl Roth, Karlsruhe, Germany) in PBS for 30 min at 4 °C. Afterwards cells were washed twice with PBS and stored in PBS at 4 °C until histological staining. Directly before staining cells were washed three times with deionized water (Millipore, Darmstadt, Germany).

Lipid droplets were stained with *Oil Red O:* The dye was dissolved at 0.5% (*w*/*v*) in 60% (*v*/*v*) 2-propanol and the solution was filtered. Cells were stained with this solution for 30 min in the dark. The staining solution was removed by three washes with water (Millipore quality).

Calcium ions were stained with *Alizarin Red S:* Cells were stained with a 1.0% (*w*/*v*) aqueous Alizarin Red S (Carl Roth) solution (pH = 4.2, made in-house) for one hour at room temperature. After this, the staining solution was removed by three washes with water (Millipore quality).

### 2.9. Quantification of Calcium and Phosphate Ions in the Cell Layer

Cells were fixed in 4% (*w*/*v*) paraformaldehyde in Tris-buffered saline solution (TBS) for 30 min, washed twice in TBS, and harvested in 1 M HCl using a cell scraper. This mix was incubated at 37 °C for 48 h. Cell debris were removed by centrifugation and the supernatants were collected and stored at −20 °C. The subsequent quantification of calcium and phosphate ions in the cell layer was carried out using the Calcium (CPC) LiquiColor^®^ Test and the QuantiChrom^TM^ Phosphate Assay, respectively. Both tests were carried out according to the protocols provided by the respective manufacturer (Stanbio, Boerne, USA and Gentaur Molecular, Aachen, Germany).

### 2.10. Determination of Glycosaminoglycan-to-DNA Ratio for Chondrogenic Differentiation

The chondrogenic pellets (one pellet per experimental condition) were digested at least 18 h at 60 °C with 0.05 mg/mL papain in 100 mM phosphate buffer containing 10 mM EDTA and 10 mM cysteine, pH 6.5. The DNA was stained with 0.2 µg/mL aqueous solution Hoechst 33258 (Sigma) and the fluorescence was measured immediately with a plate reader (excitation 360 nm; emission 460 nm). After staining of glycosaminoglycans with 0.1 mg/mL 1,9-dimethyl-methylene blue (Sigma-Aldrich; 5 mg 1,9-dimethyl-methylene blue mixed with 152 mg Glycin, 119 mg NaCl, and 4.75 mL 0.1 M HCl and filled up with water to 50 mL) the absorbance was read with a plate reader at 530 nm. DNA and glycosaminoglycan content were measured from the same pellet. The ratio of glycosaminoglycan and DNA was calculated by Excel.

### 2.11. Transcriptome Analyses

Cells from three different donors (C, D, and F) were randomly chosen. The conditions no heparin and high heparin (called “cell pairs” in the manuscript) were used and RNA obtained at day 0 before induction of osteogenic differentiation was subjected to the analyses.

The Microarray utilized in this study represents a refined version of the Whole Human Genome Oligo Microarray 4 × 44K v2 (Design ID 026652, Agilent Technologies), called “026652QM_RCUG_HomoSapiens” (Design ID 084555) developed by the Research Core Unit Genomics (RCUG) of Hannover Medical School. Microarray design was created at Agilent’s eArray portal using a 1 × 1 M design format for mRNA expression as template. All non-control probes of design ID 026652 have been printed five times within a region comprising a total of 181560 Features (170 columns × 1068 rows). Four of such regions were placed within one 1 M region giving rise to four microarray fields per slide to be hybridized individually (Customer Specified Feature Layout). Control probes required for proper Feature Extraction software operation were determined and placed automatically by eArray using recommended default settings. The array contained 34,127 probes covering a total of 25,992 genes.

100 ng of total RNA were used as input. Synthesis of Cy3-labeled cRNA was performed in ¾ reaction volumes with the “Low Input Quick Amp Labeling Kit One-Color” (#5190-2305, Agilent Technologies) according to the manufacturer’s recommendations. cRNA fragmentation, hybridization and washing steps were carried out as recommended in the “One-Color Microarray-Based Gene Expression Analysis Low Input Quick Amp Labeling Protocol V6.7”, except that 2500 ng of labeled cRNA were used for hybridization.

Slides were scanned on the Agilent Micro Array Scanner G2565CA (pixel resolution 3 µm, bit depth 20).

Data extraction was performed with the “Feature Extraction Software V10.7.3.1” by using the recommended default extraction protocol file “GE1_107_Sep09.xml”.

Measurements of on-chip replicates (quintuplicates) were averaged using the geometric mean of processed fluorescence intensity values of the green channel, “gProcessedSignal” (gPS) to retrieve one resulting value per unique non-control probe. Single Features were excluded from averaging, if they i) were manually flagged, ii) were identified as Outliers by the Feature Extraction Software, iii) lay outside the interval of “1.42× interquartile range” regarding the normalized gPS distribution of the respective on-chip replicate population, or iv) showed a coefficient of variation of pixel intensities per Feature which exceeded 0.5.

Averaged gPS values were normalized by global linear scaling: All gPS values of one sample were multiplied by an array-specific scaling factor. This factor was calculated by dividing a “reference 75th Percentile value” (set as 1500 for the whole series) by the 75th Percentile value of the specific Microarray to be normalized (“Array I” in the formula shown below). Accordingly, normalized gPS values for all samples (microarray data sets) were calculated by the following formula:

normalized gPSArray i = gPSArray i × (1500/75th PercentileArray i)(2)

Finally, a lower intensity threshold (surrogate value) was defined based on intensity distribution of negative control features. This value was fixed at 15 normalized gPS units. Those measurements which fell below this intensity cutoff were substituted by the respective surrogate value of 15.

Fold changes were calculated as sample with heparin per sample without heparin for the three cell preparations. Genes were selected for verification by quantitative realtime PCR by the following criteria: 1. All probes for the same gene should demonstrate at least twofold changes (up- or downregulation) for at least two cell pairs isolated in the presence of heparin compared to the preparations in the absence of heparin. 2. In case of a smaller fold change, the change for the third cell pair had to be up- or downregulated like for the other two cell pairs. Here, the mean change (expressed as geometric mean of the three ratio values) had to be more than twofold. 3. For downregulated genes, the normalized gPS values had to be >100 for the samples without heparin (i.e., were lower in the samples with heparin). 4. For upregulated genes, the normalized gPS values had to be >100 for the samples with heparin (i.e., were lower in the samples without heparin). The conditions 3 and 4 were chosen to get rid of less reliable measurements nearby the sensitivity limit of the microarray assay. The data discussed in this publication have been deposited in NCBI’s Gene Expression Omnibus (Edgar et al., 2002) and are accessible through GEO Series accession number GSE145433 (https://www.ncbi.nlm.nih.gov/geo/query/acc.cgi?acc=GSE145433).

### 2.12. Quantitative Realtime RT-PCR

qRT-PCR analyses were performed by using the Applied Biosystems^®^ StepOnePlus instrument (Life Technologies, via Thermo Fisher Scientific). Each sample was measured in duplicates and the mean values were calculated. The gene specific assays as well as the Fast Advanced Mastermix were purchased from Life Technologies: *RPS29* (Hs03004310_g1; house-keeping gene), Tissue Non-Specific Alkaline Phosphatase (*ALPL*, Hs00758162_m1), Osteocalcin (*BGLAP*, Hs01587814_g1), Bone Sialoprotein (*IBSP*, Hs00173720_m1), Runt-Related Transcription Factor (*RUNX2*, Hs00231692_m1), Osteopontin (*SPP1*, Hs00959010_m1) (all for osteogenic differentiation), Aggrecan (*ACAN*, Hs00153936_m1), Collagen Type II Alpha I (*COL2A1*, Hs01064869_m1), Sex Determining Region Y-Box 9 (*SOX9*, Hs00165814_m1), Matrix Metalloprotease 13 (*MMP13*, Hs00233992_m1) (all for chondrogenic differentiation), peroxisome proliferator- activated receptor gamma (*PPARG*, Hs01115513_m1), Fatty Acid Binding Protein 4 (also called adipocyte-FABP or adipocyte protein 2 (aP2), gene name *FABP4*, Hs00609791_m1) (both for adipogenic differentiation). Amphiregulin (*AREG*, Hs00950669_m1), Angiotensinogen (*AGT*, Hs01586213_m1), Calponin, basic (*CNN1*, Hs00959434_m1), C-C motif chemokine ligand 13 (*CCL13*, Hs00234646_m1), *CD36* (Hs00354519_m1), C-X-C motif chemokine ligand 3 (*CXCL3*, Hs00171061_m1), C-X-C motif chemokine ligand 14 (*CXCL14*, Hs01557413_m1), Forkhead Box Q1 (*FOXQ1*, Hs00536425_s1), Matrix Gla Protein (*MGP*, Hs00179899_m1), serpin peptidase inhibitor clade A3 (*SERPIN A3*, Hs00153674_m1), and Secreted Frizzled-Related Protein 4 (*SFRP4*, Hs00180066_m1) were analyzed as potentially heparin-regulated genes. Analyses were implemented according to the manufacturer´s instructions. Data were evaluated by the delta C_T_-method with ∆C_T_ = C_T_ (cycle threshold, gene of interest) minus C_T_ (cycle threshold, housekeeper, here *RPS29*) resulting in relative gene expressions of 2^-∆CT^.

## 3. Results

### 3.1. Many BM-MSC Preparations Exhibit Weak In Vitro Differentiation Potential

In our laboratory, we have isolated MSCs from bone marrow of more than 100 donors. The material was obtained upon implantation of total hip arthroplasty during preparation of the femoral canal. The underlying diagnosis of the patients was progressive primary or secondary osteoarthritis of the hip. Exclusion criteria were (bone) tumors, diseases of the bone marrow (e.g., leukemia) or past treatments which possibly could affect the bone marrow. To isolate the MSCs, the heparin-anticoagulated bone marrow was diluted with PBS and processed as described in Materials and Methods. We routinely characterize randomly chosen cell samples by flow cytometry and in vitro differentiation assays using standard methods (compare also Materials and Methods), in particular for osteogenesis and chondrogenesis.

**Figure 1:**
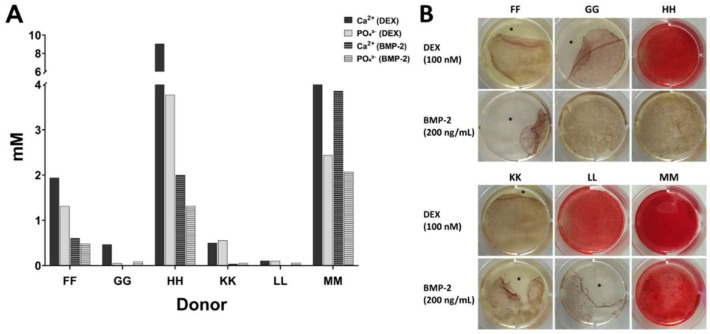
Osteogenic differentiation in vitro. Differentiation was performed for 27 days as described in Materials and Methods (cf. Supplemental Table S1 for information on donors). (**A**) Quantification of calcium ions and phosphate ions. (**B**) Alizarin Red S-staining to detect calcium ions in the cell layer. Some wells demonstrate cell layers which detached during the staining procedure (denoted by asterisks).

During these studies we noticed that many cell preparations exhibited low osteogenic and chondrogenic differentiation potential. These findings were obtained by cytochemical staining, complemented by the analysis of marker gene expressions for the respective differentiation lineages. Accessory methods included determination of the calcium-to-phosphate ion ratio in the cell layers for in vitro osteogenesis and the determination of glycosaminoglycans for in vitro chondrogenesis. Representative data for MSCs from six donors which were analyzed in parallel for their in vitro osteogenic differentiation potential by induction with 100 nM dexamethasone or with 200 ng/mL bone morphogenetic protein-2 (BMP-2) plus 50 µM ascorbate-2-phosphate and 10 mM beta-glycerophosphate are shown in Figure 1.

Figure 1A displays the results of the quantitative assessment of calcium and phosphate ions in the cell layers. MSCs from donors HH and MM showed notable accumulations (arbitrarily defined as ion concentrations >1 mM) of calcium and phosphate ions in the cell layers with both osteogenic induction protocols. For donor FF, concentrations >1 mM for both ions were only achieved by dexamethasone induction. For donors GG, KK, LL, all ion concentrations were <1 mM independent of the induction protocol. Figure 1B shows the results of Alizarin Red S-staining for calcium ions in the cell layer. This was clearly positive (intense red color) for 3 donors (HH, LL, MM) using dexamethasone. Only one donor (MM) showed a clear positive Alizarin Red S-staining after induction by BMP-2. Comparing results from concentration measurements and staining, MSCs from donor FF deposited calcium and phosphate ions in the cell layer particularly in the presence of dexamethasone (>1 mM) whereas Alizarin Red S-staining was unremarkable. MSCs from donors GG and KK showed low ion concentrations and negative staining with both induction protocols. MSCs from donor HH deposited calcium and phosphate ions >1 mM in the cell layer with both induction protocols but in the presence of BMP-2, Alizarin Red S-staining was unremarkable. On the contrary, for MSCs from donor LL, the positive Alizarin Red S-staining with dexamethasone as inducing agent could not be substantiated by quantification of calcium and phosphate ions in the cell layer. Only MSC from donor MM gave consistently positive readouts for both induction protocols, evidenced both by the concentration determinations and the staining. Figure 1B also demonstrates that some cell layers partially detached during the staining procedure (marked by asterisk).

Supplemental Figure S1 shows that in more cell preparations, eleven in total, only one Alizarin Red S-staining was clearly positive with the dexamethasone-containing osteogenic induction protocol. Representative flow cytometric data for four randomly selected cell preparations are shown in Supplemental Figure S2. CD14, CD19, and CD45 were found to be absent from the cell preparations compliant with the minimal criteria [1,2] with the exception of one donor (donor MM: 8.01% CD14). HLA-DR was expressed at a level of 64% to 86% which is due to the presence of FGF-2 in the growth medium [17]. CD73, CD90, and CD105 were also expressed on the cell surface with CD73 exhibiting more or less 100% expression, CD105 amounting to more than 90% and CD90 reaching a maximum of 73%.

We therefore hypothesized that heparin might impede the biological function (as assessed by in vitro differentiation into the osteogenic, adipogenic and chondrogenic lineages) of isolated cells. The results of detailed investigations are presented in the following sets of data.

### 3.2. Choice of Patients for Bone Marrow Sampling in the Absence or Presence of Heparin

Twelve donors, A–L, were chosen for inclusion in the study based on their good physical condition (ASA status classification I or II). One patient suffered from von Willebrand-disease and needed substitution therapy. Patient information including comorbidities is included in Table 1. None of the patients received medication for hemodilution preoperatively. Thrombosis prophylaxis was started only postoperatively. Thus, in none of the patients thrombosis prophylaxis affected the collected bone marrow specimens. In case of increased intraoperative bleeding (four patients) tranexamic acid was given intraoperatively (Table 1).

Bone marrow was collected from these twelve patients during preparation of the femoral cavity for the implantation of the femoral stem for a total hip arthroplasty. Bone marrow was collected intraoperatively as explained in more detail in the Materials and Methods section. During this process, no coagulation was observed. For each donor, three aliquots of 5 mL bone marrow were collected: Condition 1 with no heparin in the syringe, condition 2 supplemented with 1.5 mL heparin (i.e., 1500 IU/mL bone marrow) and condition 3 supplemented with 3.5 mL heparin (i.e., 3500 IU/mL bone marrow). The quality of bone marrow was assessed intraoperatively macroscopically and graded from 1 (excellent) to 6 (inferior) (Table 2). In addition, the sequence of sample preparations was varied intraoperatively, i.e., condition 1 without heparin was not always the first sample to be prepared (Table 2) in order to avoid bias.

### 3.3. Heparin Anticoagulation Was Not Necessary for Isolation of Mononuclear Cells from Bone Marrow

**Figure 2:**
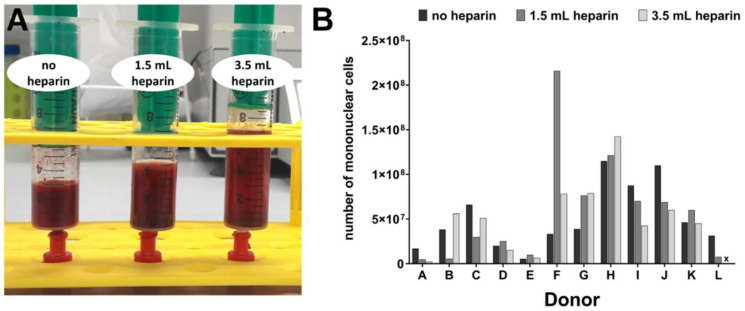
Bone marrow without and with heparin. (**A**) Representative photographs of bone marrow samples from one randomly chosen donor using the following conditions: No heparin, with 1.5 mL heparin and with 3.5 mL heparin before further processing. No 0.9% NaCl was added at this stage yet. No signs of coagulation are visible especially for the condition without added heparin. (**B**) Total numbers of mononuclear cells isolated from each sample (passage zero). x: For donor L, no mononuclear cells could be counted with the high amount of heparin. In this case, the putative cell-containing fraction was seeded into a T25 flask and plastic-adherent cells successfully grew out and could be expanded and studied in subsequent experiments.

The samples without heparin addition did not exhibit macroscopic signs of coagulation upon visual inspection at arrival in the laboratory (Figure 2A), even upon overnight storage of the sample from donor J. During the processing of bone marrow for MSC isolation, the samples from condition 1 without heparin, but always diluted with 3.5 mL 0.9% NaCl, were most easily filtrated, cf. Table 2. This confirms the finding that no substantial coagulation had taken place. It was further substantiated by quantification of the number of mononuclear cells which were isolated from the density gradients, displayed in Figure 2B.

Table 2 reveals that this cell number did not correlate with the intraoperative assessment of the bone marrow samples. One example is donor H which showed high numbers of mononuclear cells in the absence or presence of heparin but it had a relatively bad score of 5 with respect to the quality of intraoperatively sampled bone marrow (6 being the worst score). On the contrary, samples with excellent score 1 from donors B, E, and L provided relatively low numbers of mononuclear cells. In addition, the number of mononuclear cells did not correlate with the absence or presence of heparin. In particular, the cell numbers were not lower in the absence of heparin which confirmed the finding of non-existing macroscopic coagulation.

### 3.4. Heparin Anticoagulation Had No Influence on Plastic Adhesion, Morphology, and Proliferation of BM-MSCs

**Figure 3:**
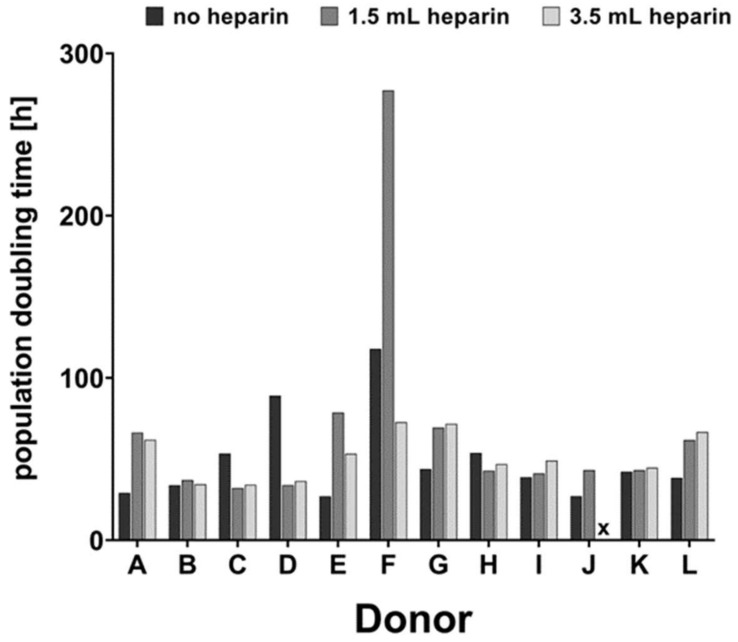
Population doubling times. Determined for individual cell preparations between passage 1 and 2. x: cells stopped to proliferate.

After isolation from the density gradient, mononuclear cells attached well to the tissue culture plastic. The morphology remained fibroblastic during subsequent passages. The population doubling time between passage 1 and passage 2 is shown in Figure 3. The mean population doubling time for cells isolated in the absence of heparin was 49.5 h, for cells isolated with 1.5 mL heparin per 5 mL bone marrow 68.9 h, and 52.0 h for cells isolated with 3.5 mL heparin per 5 mL bone marrow. Only one donor (F) showed a notably higher population doubling time with 1.5 mL heparin which was not observed in the presence of high heparin content.

### 3.5. Heparin Anticoagulation Had No Influence on the Cell Surface Presentation of Selected CD Antigens of BM-MSCs

**Figure 4:**
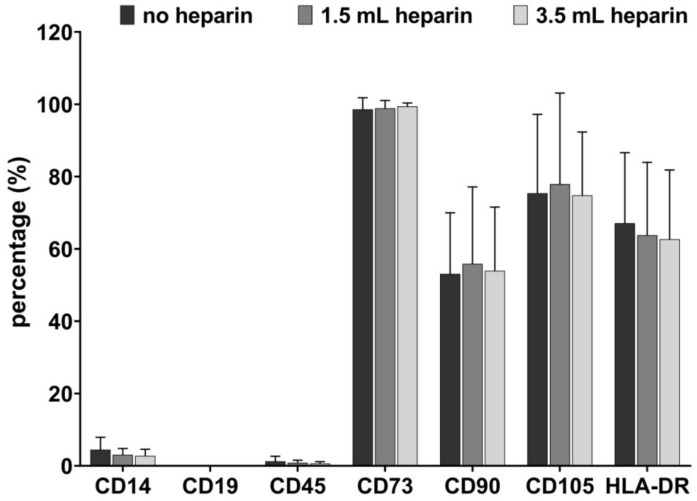
Flow cytometric data. Flow cytometry for mesenchymal stromal cell (MSC) populations derived from bone marrow in passages 2 to 4 as applicable. Arithmetic means and standard deviations for all twelve donors are shown.

Flow cytometric data of cell populations from all twelve donors are shown in Figure 4. CD14, CD19, and CD45 were largely absent whereas HLA-DR was expressed at a level of 62.66 ± 19.17% (3.5 mL heparin) to 67.14 ± 19.48% (no heparin). CD73 was expressed almost 100% on the cell surface for all donors and sample preparations. CD90 was expressed at a range between 53.09 ± 16.90% (no heparin) to 55.87 ± 21.27% (1.5 mL heparin). CD105 was expressed in a range of 74.83 ± 17.49% (3.5 mL heparin) to 77.92 ± 25.18% (1.5 mL heparin). Summarizing these data, the presence of heparin had no influence on the cell surface expression of any of the investigated CD antigens.

Due to their low proliferative capacity after passage 2 (which was independent of the addition of heparin), cells from donors B and H were just analyzed during chondrogenic differentiation and excluded from all other analyses. Donor J cells isolated with the addition of 3.5 mL heparin did not proliferate substantially after passage 3 and therefore could not be subjected to the differentiation experiments.

### 3.6. Heparin Anticoagulation Had No Influence on Adipogenic In Vitro Differentiation of BM-MSCs

**Figure 5:**
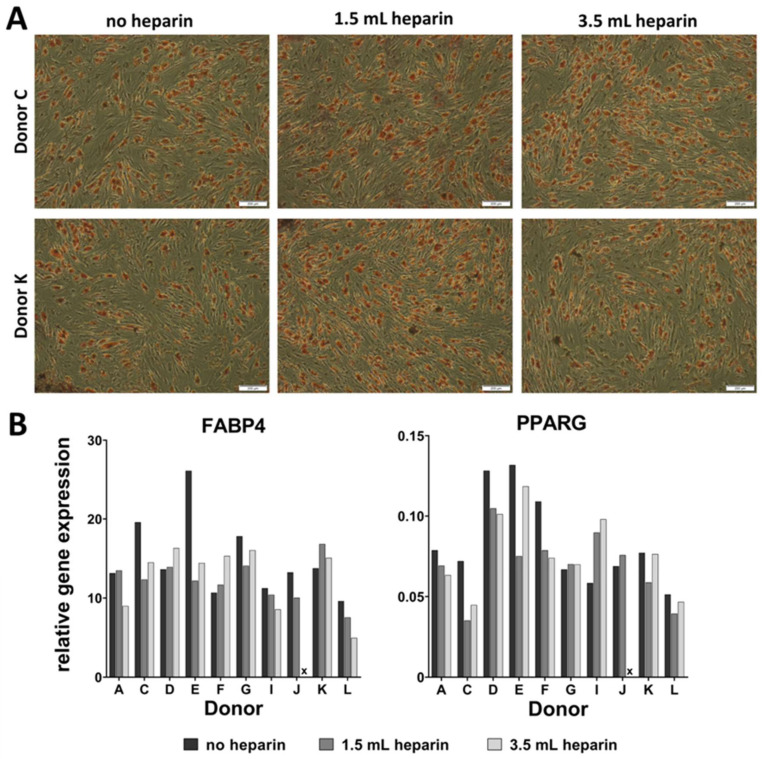
Adipogenesis in vitro. (**A**) Oil Red O-staining, microscopic views. Scale bar: 200 µm. (**B**) Relative gene expression analysis (2^−ΔCt^) for adipogenic marker genes at day 14. x: available cell numbers were too low for inclusion in the analysis.

Adipogenic induction was performed in sixwell-plates in duplicates for cells from ten out of twelve donors. Results were subjected to Oil Red O-staining at day 14 of differentiation and gene expressions were analyzed at day 0 (immediately before induction of differentiation) and at day 14 (end of differentiation). Figure 5A depicts microscopy of Oil Red O-stainings for donors C and K (Supplemental Figure S3A shows the stained sixwell-plates of all donors). There is no substantial variation in staining intensity between the cells between individual donors and between bone marrow-processing conditions with respect to heparin addition. One key transcription factor during adipogenic differentiation is *PPARG* which is early upregulated during this process. Amongst other genes, it targets *FABP4* which presents a late stage of adipogenic differentiation. FABP4 is an intracellular protein which transports lipids in adipocytes. Both genes are routinely used for assessment of adipogenic differentiation of human MSCs [18,19,20]. These genes did not show any trend with respect to inter-donor variabilities of bone marrow processing conditions (Figure 5B: relative gene expression 2^−∆Ct^ calculated versus *RPS29* as house-keeping gene).

### 3.7. Heparin Anticoagulation Had No Influence on Osteogenic In Vitro Differentiation of BM-MSCs

**Figure 6:**
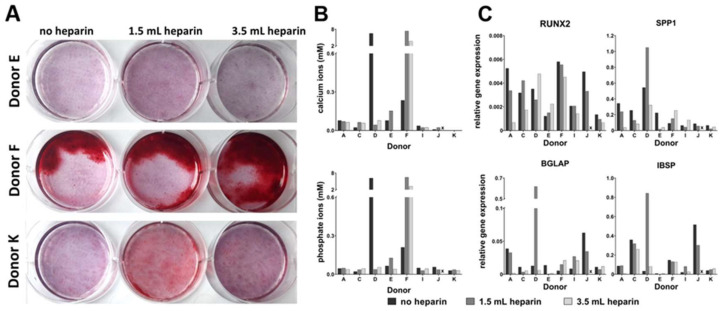
Osteogenesis in vitro. (**A**) Alizarin Red S-staining for calcium ions of randomly chosen donors (E, F, K), macroscopic views. (**B**) Determination of calcium and phosphate ion content in the cell layers as arithmetic means of individual duplicate samples. (**C**) quantitative realtime PCR (qRT-PCR) analysis for osteogenic marker genes (2^−ΔCt)^ at day 27. x: available cell numbers were too low for inclusion in the analysis.

Osteogenic induction was performed in sixwell-plates in duplicates for cells from eight out of 12 donors with human recombinant BMP-2, beta-glycerophosphate, and ascorbate. Results of differentiation were analyzed at day 27. Since cells from donor G detached at day 19, they were not included in the analyses. Cell numbers from donor L were too low to perform the experiment. Figure 6A depicts the macroscopic Alizarin Red S-stainings of three randomly selected donors. Cells from donor E demonstrated faint red color. Cells from donor F isolated in the absence (left well) or the presence (middle: 1.5 mL heparin, right: 3.5 mL heparin) of heparin showed intensely red stained regions contrasting with unstained regions. This staining pattern appeared highly conspicuous, reminiscent of a spontaneously catalyzed reaction. Donor K showed a more intense Alizarin Red S-staining with 1.5 mL heparin but less without heparin and with 3.5 mL heparin. The Alizarin Red S-staining of MSCs from the other donors are shown in Supplemental Figure S3B: Cells from donor D demonstrated intense red staining only in the absence of heparin. All other staining was faint, with no relation to the heparin amount.

Figure 6B shows the amount of calcium and phosphate ions quantified in the cell layers from separate replicate samples. The results closely mirrored the corresponding Alizarin Red S-staining in Figure 6A and Supplemental Figure S3B. Here, high amounts of calcium and phosphate ions were detected in cells from donor D in the absence of heparin and cells from donor F isolated in the presence of heparin (1.5 mL and 3.5 mL heparin). However, despite the speckled Alizarin Red S-staining of cells from donor F in the absence of heparin the amount of calcium and phosphate ions was only slightly higher than the background. No correlation between the staining or calcium and phosphate content in the cell layers with the amount of heparin was observed.

qRT-PCR analyses were performed for genes which are induced in the course of differentiation of mesenchymal stromal cells into osteoblast-like cells: Runt-Related Transcription Factor 2 (*RUNX2*), Secreted Phosphoprotein 1 (Osteopontin, gene name *SPP1*), Bone Gamma-Carboxyglutamate protein (Osteocalcin, gene name *BGLAP*), and Integrin-Binding Sialoprotein (*IBSP*). Runt-Related Transcription Factor 2 and Osteopontin are proteins which are upregulated during matrix development/maturation. Bone Gamma-Carboxyglutamate protein is upregulated in the late phase of differentiation during mineralization. Integrin-Binding Sialoprotein is a major bone matrix protein [21,22,23,24,25]. Their relative gene expressions were determined versus the house-keeping gene *RPS29* (Figure 6C). *RUNX2* relative gene expression was high for cells from donor F with the speckled Alizarin Red S-staining but lower in cells from donor D, with no heparin, with intense Alizarin Red S-staining. *SPP1* relative gene expression was high for cells from donor D but the sample with prominent expression with 1.5 mL heparin exhibited negligible Alizarin Red S-staining. The same applies for *BGLAP* and *IBSP*. Here, the relative gene expressions of both genes were high for donor D with 1.5 mL heparin but lower for the conditions without heparin or with 3.5 mL heparin. In summary, the analyzed genes did not indicate any trend with respect to inter-donor variabilities of bone marrow processing with no heparin, 1.5 mL and 3.5 mL heparin.

### 3.8. Heparin Anticoagulation Had No Influence on Chondrogenic In Vitro Differentiation of BM-MSCs

**Figure 7:**
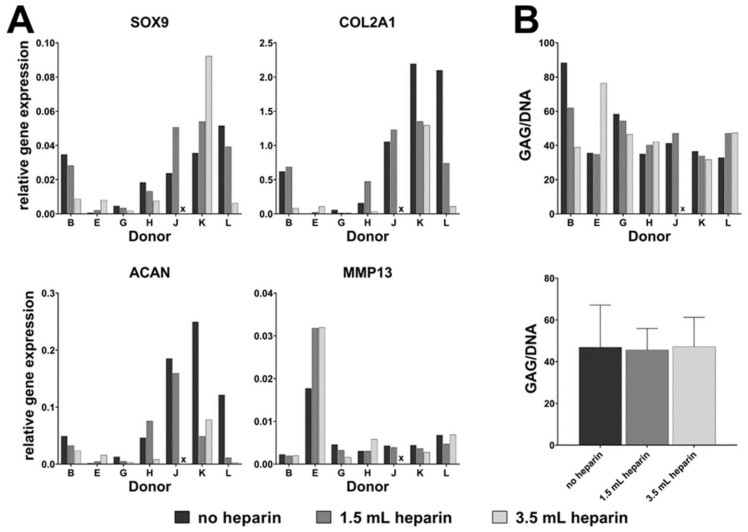
Chondrogenesis in vitro. (**A**) qRT-PCR analyses of chondrogenic marker genes (2^−ΔCt^) at day 28. (**B**) Glycosaminoglycan(GAG)/DNA ratio at day 28 (upper panel) and mean values of the GAG/DNA-ratios (lower panel) for these seven donors were comparable in all three conditions (lower panel).

Chondrogenic induction was performed in three-dimensional pellet cultures in duplicates for cells from seven out of twelve donors. Results were analyzed at day 28 of differentiation. Gene expression analyses were performed for genes expressed at different stages of chondrogenic in vitro differentiation: Sex Determining Region Y-Box 9 (*SOX9*) is expressed early during differentiation, Aggrecan (*ACAN*, Hs00153936_m1) and Collagen type II alpha I (*COL2A1*, Hs01064869_m1) are markers for mature chondrocytes, and Matrix Metalloprotease 13 (*MMP13)* is expressed in hypertrophic mineralized chondrocytes and therefore indicates progressive chondrogenesis. Figure 7A shows that none of the four genes indicated an influence of its expression on heparin. Higher gene expressions of *ACAN* were found for donors J and K with no heparin, *SOX9* for donor K with 3.5 mL heparin or *COL2A1* for donors K and L with no heparin. *MMP13* demonstrated notably higher expression levels in all three heparin conditions in cells from donor E while the expressions of *SOX9*, *ACAN* and *COL2A1* were generally lower in cells from donor E than in the other six cell populations. Since glycosaminoglycan secretion is one hallmark of mature chondrocytes, the glycosaminoglycan (GAG) to DNA ratio was determined. This ratio indicated no clear influence of heparin. It was similar in the absence or presence of heparin for cells from five donors (G, H, J, K, L), decreased in the presence of heparin in cells from donor B as well as increased in the presence of heparin in cells from donor E (Figure 7B upper panel). Mean values of the GAG/DNA-ratios for these seven donors were comparable in all three conditions (Figure 7 lower panel) and did not correlate with gene expressions.

### 3.9. Microarray-Based Transcriptome Analyses

MSCs isolated from three randomly selected donors (C, D, F) in the absence and presence of high heparin content were expanded in MSC expansion medium nearly to confluence. Total RNA was isolated from these cultures and subjected to transcriptome analyses. By and large, gene regulations were small and did not exceed more than twofold changes. However, some genes stood out with respect to four criteria (explained in more detail in Materials and Methods): A mean change of at least twofold in the presence or absence of heparin for these three pairs of preparations and normalized gPS values > 100 for the samples without heparin or >100 for the samples with heparin, respectively. In the tested donors genes which were upregulated by heparin in the tested donors according to these criteria included amphiregulin (*AREG:* Related to epidermal growth factor and transforming growth factor alpha), chemokine (C-X-C motif) ligand 3 (*CXCL3:* Role in inflammation, chemoattractant for neutrophils) and *FOXQ1* (Forkhead transcription factor, favors mesenchymal phenotype, promotes anti-senescence and migration of MSCs from human umbilical cord). Several genes were putatively downregulated by heparin: Angiotensinogen (*AGT:* precursor of all angiotensin peptides; member of the non-inhibitory serine protease inhibitor superfamily), chemokine (C-C motif) ligand 13 (*CCL13:* role in inflammation, chemotactic activity for monocytes, lymphocytes, basophils, and eosinophils, but not neutrophils), chemokine (C-X-C motif) ligand 14 (*CXCL14:* chemotactic activity for monocytes but not for lymphocytes, dendritic cells, neutrophils, or macrophages), the thrombospondin receptor (*CD36:* functions as a cell adhesion molecule; binds to collagen, thrombospondin, anionic phospholipids, and oxidized LDL), calponin 1 (*CNN1* basic, smooth muscle isoform: implicated in the regulation and modulation of smooth muscle contraction; is capable of binding to actin, calmodulin, troponin C and tropomyosin), matrix Gla protein (*MGP:* the encoded vitamin K-dependent protein—osteocalcin/matrix Gla family of proteins—is secreted by chondrocytes and vascular smooth muscle cells, and functions as a physiological inhibitor of ectopic tissue calcification), serpin peptidase inhibitor, clade A (alpha-1 antiproteinase, antitrypsin), member 3 (*SERPINA3:* plasma protease inhibitor and member of the serine protease inhibitor class) and secreted frizzled-related protein 4 (*SFRP4:* contains a cysteine-rich domain homologous to the putative Wnt-binding site of Frizzled proteins; acts as soluble modulator of Wnt signaling). In summary, several of these genes like the chemokines encode secreted proteins or proteins related to cell migration. Others are transcription factors like FOXQ1 [26,27].

### 3.10. Confirmatory Expression Analyses of Genes Regulated by Heparin

**Figure 8:**
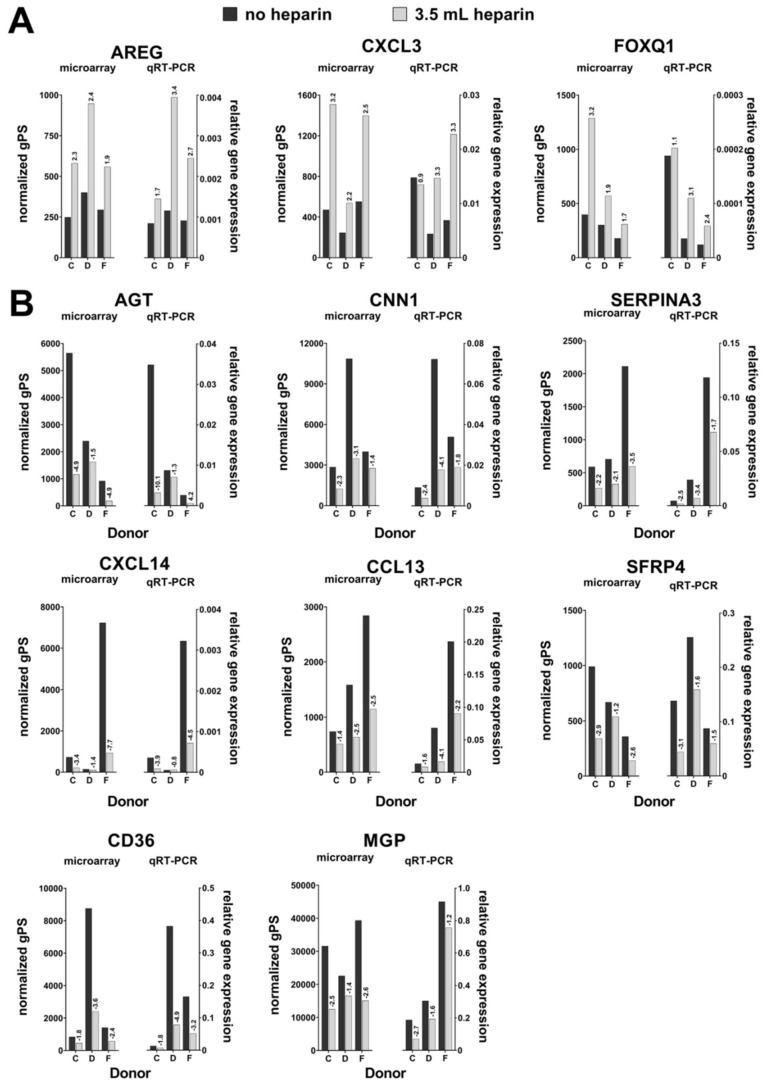
Results of comparative microarray-based transcriptome versus qRT-PCR analysis of cells from donors C, D and F. Normalized green processed signals (gPS) and qRT-PCR-based relative gene expression levels (2^−ΔCt^) of all eleven genes identified as regulated by heparin are depicted. (**A**) Relative gene expression changes of upregulated genes. (**B**) Expression changes of downregulated genes. In cases with more than one available microarray probe per gene, values of that probe with the highest mean gPS value across all tested conditions was chosen for this figure.

The three upregulated genes (*AREG*, *CXCL3,* and *FOXQ1*) and the eight downregulated genes (*AGT*, *CCL13, CD36*, *CNN1*, *CXCL14*, *MGP*, *SERPINA3*, *SFRP4*) were further investigated by qRT-PCR analysis with respect to their expression in cells from all nine donors and in consideration of all three experimental conditions. The data were normalized against the housekeeping gene *RPS29* and are displayed in Supplemental Figure S4. Figure 8 shows a comparison of the data obtained by microarray-based transcriptome analyses and qRT-PCR for the donors C, D, and F. Twofold changes in gene expression between cell samples without heparin compared to cell samples isolated with the addition of 3.5 mL heparin were considered as “regulated” in these donors.

Figure 8 demonstrates an overall high degree of consistency of the results of the microarray and qRT-PCR analyses for up- or downregulated genes in cells isolated from 3.5 mL heparin compared to untreated cells. This concordance in mRNA expression changes largely comprises all three selected donors C, D, and F but with one notable exception for the upregulated genes: donor C who did not show an upregulation of the *CXCL3* gene in qRT-PCR. Supplementary Figure S4 adds information for donors A, E, G, I, K, and L and shows that the upregulation of *AREG*, *CXCL3,* and *FOXQ3* could not be confirmed for all analyzed donors. In particular, two donors (K and L) showed even a decrease in expression levels with the addition of 3.5 mL heparin.

The downregulation or a trend towards lower expression of *AGT*, *CCL13, CD36*, *CNN1*, *CXCL14*, *MGP*, *SERPINA3*, and *SFRP4* in cells isolated with the addition of 3.5 mL heparin could be confirmed not only for the donors C, D, and F but also for the additional six donors with few exceptions such as donor A, *AGT* (undetectable expression levels in the absence of heparin and with 3.5 mL heparin) as well as *CD36* and *CNN1* or donor E, *CNN1* (Supplemental Figure S4).

Interestingly, gene expression levels in cells isolated from bone marrow with 1.5 mL heparin often did not show intermediary gene expression as might be expected compared to expression in cells without or with the high amount of heparin (Supplemental Figure S4). As one striking example, *FOXQ1* relative expression for donor K is highest with 1.5 mL heparin (2.4 fold higher than without heparin). As another striking example, *MGP* expression is lowest for donors C, E, F, and K with 1.5 mL heparin.

Overall, even though a high degree of methodical concordance could be demonstrated, taken all donors and heparin concentrations into account, no single gene exhibited a clear and consistent tendency of heparin-dependent regulation (Figure 8).

## 4. Discussion

In the field of mesenchymal stromal cells, diverse cell populations from many different tissues may be summarized due to their joint minimal features. In the present study, the cell populations were defined as bone marrow-MSCs based on their plastic-adherent growth, the presence or absence of cell surface antigens determined by flow cytometry and their in vitro differentiation. Of special notice, all cell preparations were striking with respect to their CD90 expression, which was notably less than the 95%, which is suggested by the minimal criteria for positive antigens on the MSC surface. We have observed this low level of CD90 surface expression with antibodies against different CD90 epitopes and have also observed that the surface expression of CD90 declines with passaging [28]. This low CD90 surface expression seems to be favored by the medium used for MSC expansion since it was observed to much less extent in media containing human serum or human platelet lysate and in the absence of FGF-2 (unpublished data).

It becomes more and more relevant that more precise definitions will be necessary to further develop the field in the future. A prominent example from the present study is the cytochemical staining of osteo- or adipogenic differentiation as well as the glycosaminoglycan/DNA ratios of chondrogenic differentiation, which did not correlate with the results of gene expression analyses of the representative marker genes. Only the elevated ratio of calcium to phosphate in the cell layers—indicative of mineralization in form of hydroxyapatite [29]—correlated with positive Alizarin Red S-staining since both methods quantify calcium ions. However, it is important that cell layers do not detach during investigation of the cell culture. In many cases—in the present study as well as in studies from other groups—floating material consisting of calcium phosphate layers was removed by medium changes during cell culture and prevents accurate determinations of the calcium content. Finally, it is not clear whether faint Alizarin Red S-staining should be taken as evidence for successful differentiation—as with the frequency of washing after removing the stain the intensity declines.

Our data demonstrate that the addition of heparin for anticoagulation of bone marrow during sampling has no influence on the quality of isolated MSCs judged by the minimal criteria and transcriptome analyses. Cells isolated in the absence of heparin and in the presence of high heparin concentrations likewise adhered to tissue culture material as well as no effect during proliferation was observed (assessed by population doubling times). Expression of the cell surface antigens according to the International Society for Cell Therapy minimal criteria were not affected by heparin and neither was the outcome of in vitro trilineage differentiation. Transcriptome analyses were performed in order to obtain a more detailed molecular understanding of the effect of heparin on the isolated cells. Consistent changes of twofold or more were observed in eleven genes only while 25,992 genes were present on the arrays, and the changes in expression levels must be considered as relatively low. These genes were verified by qRT-PCR analyses for nine cell populations with all experimental conditions. Overall, across all tested donors no single gene exhibited a clear tendency of heparin-dependent regulation (Supplemental Figure S4). These findings deviate from those of earlier publications. However, it is important to remember that in the present study, the effect of heparin addition during bone marrow sampling on subsequent cell performance was assessed; no heparin was added during cell expansion. In the previous studies, isolated cells were expanded and cultured in the presence of heparin, which is a different approach. The results of the previous studies were briefly summarized in the introduction [11,12,14].

Several more genes were detected in the transcriptome analysis, which were regulated in only two of the three investigated donor cell pairs. Therefore, these genes were not verified by qRT-PCR analyses. Anyhow they might be potentially interesting due to the fact that some of these genes function in blood coagulation, which seems reasonable in the context of heparin as a stimulus: Thrombomodulin (upregulated), haptoglobin, and transferrin (downregulated with increasing heparin concentration).

The hemocompatibility of culture-expanded MSCs is an important issue, in particular in in vivo applications using systemic administration. The instant blood-mediated inflammatory reaction (IBMIR) is responsible for compromising the survival and function of systemically applied cells [30,31]. In contrast, endothelial cells are largely protected from this reaction since they have strong antithrombotic properties mediated by expression of nitric oxide, prostacyclin, tissue factor pathway inhibitor (gene name: *TFPI*), and heparin-bound antithrombin [31]. One study comparing gene expression in human MSCs and endothelial cells could demonstrate that tissue factor pathway inhibitor (gene name: *TFPI*), tissue-type plasminogen activator (gene name: *PLAT*), urokinase-type plasminogen activator (gene name: *PLAU*), prostacyclin synthase (gene name: *PTIGS*, with prostacyclin 2, PGI2, being a strong inhibitor of platelet activation), thrombomodulin (gene name: *THBD*), plasminogen activator inhibitor type 1 (gene name: *SERPINE1*), and procoagulant platelet factor 4 (gene name: *CXCL4*) were similarly expressed in both cell types [31]. However, strong expression of collagen type I and fibronectin 1 plus weak expression of tissue factor (also called CD142, gene name *F3*) on MSCs caused platelet activation and IBMIR [31]. Consequently, selection of tissue factor-deficient MSCs from bone marrow has been suggested to improve hemocompatibility [32].

Some of the bone marrow donors in the present study had pre-existing conditions. However, except for one patient (donor K), who had a von Willebrand-syndrome, none of these comorbities is highly likely to affect the coagulation (Table 1). As pointed out in the introduction, different anti-coagulants are available, which could differentially impact on the behavior of MSCs. Consequently, an analysis comparing different anticoagulants might be performed in the future. In addition, many studies have demonstrated the decisive influence of cell expansion and culture protocols on MSC features. To mention a few: The influence of FCS or FGF-2 (which interacts with heparin). The issue of medium additives is covered in many articles (see, e.g., [15]). As a matter of fact, apart from composition, concentrations are important. We used a low concentration of 2 ng/mL FGF-2 (compared to 10 or even 20 ng/mL), which has been demonstrated to enhance the proliferative potential [33], to suppress cellular senescence [34] and, in these low concentrations, to retain the multilineage differentiation potential of MSCs [35,36].

Unfortunately, apart from adhesion to tissue culture plastics, there is no consensus for unequivocal identification and prospective isolation of MSCs, which would be highly important, e.g., the use of CD146-positive cells [37], perivascular stromal/stem cells [38], or the human skeletal stem cell characterized by surface expression of Podoplanin, CD73 (Ecto-5’-Nucleotidase) and CD164 (Endolyn) [39]. Also, MSCs isolated from different tissue sources may have a different developmental origin [40] and do not necessarily have equivalent biological properties [4,41]. The removal of the cells from their original tissue or stem cell niche is an important issue to be covered in future studies since during cell culture, the cells will lose some or many of their important features, which they exhibited in vivo. This also includes their differentiation potential which may notably differ in vitro and in vivo (see, e.g., [4,42], or opinion article [43]). Therefore, finding a way to expand MSCs in high quality with high potential for the desired application is highly important. The criterion of plastic adherence implies that additional cell types with plastic adherent properties might be co-isolated and co-expanded, e.g., macrophages or fibroblasts. One previous study from the present group of researchers has demonstrated that in passage 2 and thereafter, macrophages are not present [28]. The situation is more complicated with fibroblasts since fibroblasts and MSCs share a lot of surface markers. Consequently, it is important to find some markers, which are only expressed on the surface of MSCs. One group of researchers showed that CD146 was only present on MSCs and CD166 was upregulated in MSCs but not in fibroblasts. Another marker is CD9, which is downregulated in MSCs but upregulated in fibroblasts. However, the authors concluded that “markers distinguishing mesenchymal stem cells from fibroblasts are downregulated with passaging” [44].

The combination of flow cytometric analysis and high-throughput single cell RNA-sequencing, without in vitro-expansion of cell populations, has recently led to new insights into fibroblast populations. In fact, the fibroblast that was historically described as a connective tissue-cell that secreted proteins, especially collagen, thereby contributing to formation of the extracellular matrix of connective tissue and expressing vimentin [45], is now revealed as a population of cells with different features. Impressive studies have been conducted on fibroblasts from synovial tissue. In the literature, these cells are often termed “type B synovial lining cells”, fibroblast-like synoviocytes, mesenchymal stromal cells, or other with expression of cell surface markers like CD44 and InterCellular Adhesion Molecule-1, which characterizes these cells into the fibroblast lineage. Specifically, in the lining layer of the synovial tissue, fibroblasts were identified by expression of Fibroblast Activation Protein Alpha [46] or expression of CD55 [47]. In the sublining layer, fibroblasts were identified by expression of CD90/THY1 and additional factors [46,47] including Podoplanin [39]. Notably, CD90 is one important marker for MSCs according to the minimal criteria [1]. Similar to MSCs, studies have shown in vitro-differentiation into mesenchymal cell lineages and also described immunomodulatory properties for fibroblasts (reviewed in [45]).

## 5. Conclusions

The obtained results suggest that heparin is not necessary for anticoagulation since bone marrow samples without heparin did not coagulate and were well filtrated after dilution. In addition, mononuclear cell retrieval from the density gradient was comparable to bone marrow samples supplied with heparin. No influence on morphology, proliferation, expression of cell surface antigens or in vitro differentiation could be observed. Transcriptome analyses indicated modest changes in the expression of several genes related to coagulation or with other biological functions but showed no consistency across all tested donors.

Consequently, the use of heparin for anticoagulation of bone marrow before isolation of MSCs is not detrimental to those in vitro functions addressed by the minimal criteria as assessed by the present study. Interestingly, for patients who are under long-term systemic heparin-anticoagulation therapy there might be another question to answer: Does systemic heparin-anticoagulation therapy over a longer period of time have an effect on MSCs? Therefore, it would be interesting to analyze MSCs from such patients whether they have changes in specific functions before and after start of the anticoagulation therapy or compare these to donors without such treatment. In addition, it might be interesting to investigate the features of MSCs isolated from bone marrow after the use of other anticoagulants. Finally, with improved protocols for cell isolation and expansion, some important functional questions in the field of MSC biology will need to be re-assessed.

## Figures and Tables

**Table 1:** Information of bone marrow donors and passages of cells used in the analyses.

Donor	Gender [m/f]	Age	Body Weight [kg]	Body Size [cm]	Pre-Existing Conditions	ASA Physical Status Classification	Intraoperative Treatment with Tranexamic Acid
A	m	67	50	162	Mitral valve insufficiency II°	II	no
B	f	62	65	182	Morbus Bechterew	I	no
C	f	62	90	163	Arterial hypertension	II	yes
D	f	44	48	158	---	I	yes
E	f	79	67	160	Arterial hypertension	II	no
F	f	47	67	165	---	I	yes
G	m	37	92	186	Arterial hypertension	I	no
H	f	77	64	165	Asthma bronchiale	II	no
I	m	60	71	157	---	I	no
J	f	46	110	170	---	II	yes
K	m	59	106	180	von Willebrand-syndrome 2A → substitution; Arterial hypertension	II	no
L	m	64	70	169	Arterial hypertension; Benign hypertrophy of the prostate gland	II	no

**Table 2:** Information of bone marrow spiking with heparin.

Donor	Intraoperative Assessment of Bone Marrow Quality	Sequence of Sample Generation	Filtration of Bone Marrow Samples through 100 µm-Cell Strainer Before Density Gradient	Number of Mononuclear Cells for Sample 1 (No Heparin)	Type of Endoprosthesis Provided to Donor
A	3	2, 3, 1	1 > 3 > 2	1.688 × 10^7^	straight
B	1	3, 1, 2	1 > 2; 3	3.825 × 10^7^	straight
C	3	1, 2, 3	1 > 2; 3	6.600 × 10^7^	straight
D	4	2, 3, 1	1 > 3 > 2	2.000 × 10^7^	short
E	1	3, 1, 2	1 > 2; 3	0.525 × 10^7^	straight
F	5	3, 1, 2	1 > 2 > 3	3.325 × 10^7^	short
G	3	1, 2, 3	1 > 3 > 2	3.875 × 10^7^	straight
H	5	3, 2, 1	1; 2; 3 similar (difficult)	11.500 × 10^7^	straight
I	5	2, 1, 3	1; 2; 3 similar (easy)	8.750 × 10^7^	short
J	3	3, 2, 1	1 > 2 > 3	11.000 × 10^7^	straight
K	2	1, 2, 3	1 > 3 > 2	4.625 × 10^7^	straight
L	1	1, 2, 3	1; 2; 3 similar (easy)	3.125 × 10^7^	straight

Grading of bone marrow quality intraoperatively from 1 (excellent) to 6 (inferior); straight: straight stem prosthesis; short: short shaft prosthesis; Sample 1: 5 mL bone marrow without heparin (+ 3.5 mL 0.9% NaCl); Sample 2: 5 mL bone marrow with 1.5 mL heparin (+ 2.0 mL 0.9% NaCl); Sample 3: 5 mL bone marrow with 3.5 mL heparin (no addition of 0.9% NaCl); “>”: better than; The number of mononuclear cells for no heparin, 1.5 mL heparin and 3.5 mL heparin are displayed in Figure 2B; Donor J: no cells could be expanded with the addition of 3.5 mL heparin beyond passage 2.

## Supplementary Materials

The following are available online at https://www.mdpi.com/2073-4409/9/7/1580/s1, Supplemental Figure S1 (related to Figure 1). Osteogenic differentiation *in vitro*. Differentiation was performed for 27 days with 100 nM dexamethasone as described in Materials and Methods (cf. Supplemental Table S1 for information on donors). Alizarin Red S-staining to detect calcium ions in the cell layer. Supplemental Figure S2 (related to Figure 1). Flow cytometric data of four representative MSC populations characterized by osteogenic differentiation in Figure 1 and Supplemental Figure S1. Supplemental Figure S3 (related to Figure 5 and Figure 6). Results of *in vitro* differentiation. (A) Photos of Oil Red O-stained cell layers in sixwell-plates after 14 days of adipogenic differentiation. (B) Photos of Alizarin Red S-stained cell layers in sixwell-plates after 27 days of osteogenic differentiation. Supplemental Figure S4 (related to Figure 8). Quantitative realtime PCR analysis for genes selected from the results of transcriptome analyses. (A) Relative gene expressions (2^−ΔCt^) of three genes identified to be upregulated by heparin. (B) Relative gene expressions (2^−ΔCt^) of nine genes identified to be downregulated. Cells from donors C, D and F had been investigated by the transcriptome analyses. Supplemental Table S1: Information on bone marrow donors and passages of cells used in the analyses depicted in Figure 1 and Supplemental Figures S1 and S2. n. a.: not available.
