# Supplementary material for: Heparin Anticoagulant for Human Bone Marrow Does Not Influence In Vitro Performance of Human Mesenchymal Stromal Cells
Source: Cells. 2020 Jun 29;9(7):1580. doi: 10.3390/cells9071580 (PMC7408646; doi:10.3390/cells9071580)
Supplement: Supplementary file 1 [file cells-09-01580-s001.pdf]

Supplemental Material

Supplemental Table S1: Information on bone marrow donors and passages of cells used in the analyses depicted in Figure 1 and Supplemental Figures S1 and S2. n. a.: not available

Donor	Gender (m/f)	Age
AA	f	42
BB	m	49
CC	m	36
DD	m	52
EE	m	21
FF	f	69
GG	f	74
HH	f	81
II	f	45
JJ	m	60
KK	m	63
LL	m	79
MM	f	76
NN	m	79
OO	f	72
PP	f	71
QQ	m	n. a.
RR	f	48
SS	f	41

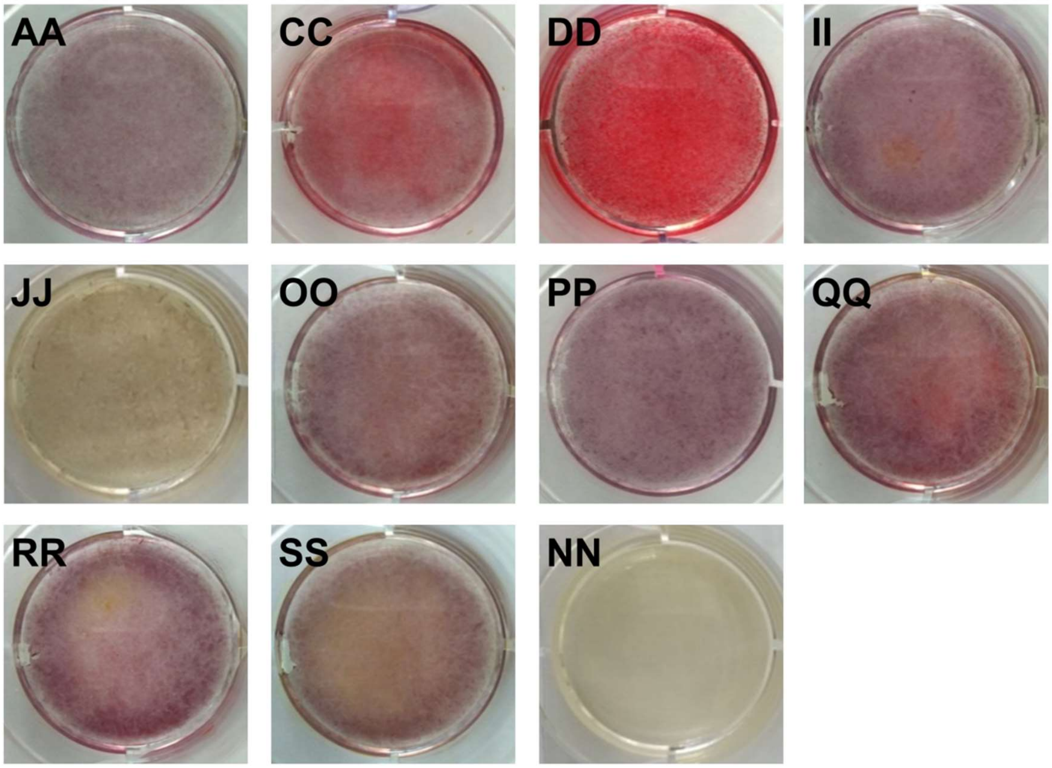

Supplemental Figure S1 (related to Figure 1). Osteogenic differentiation *in vitro*. Differentiation was performed for 27 days with 100 nM dexamethasone as described in Materials and Methods (cf. Supplemental Table S1 for information on donors). Alizarin Red S-staining to detect calcium ions in the cell layer.

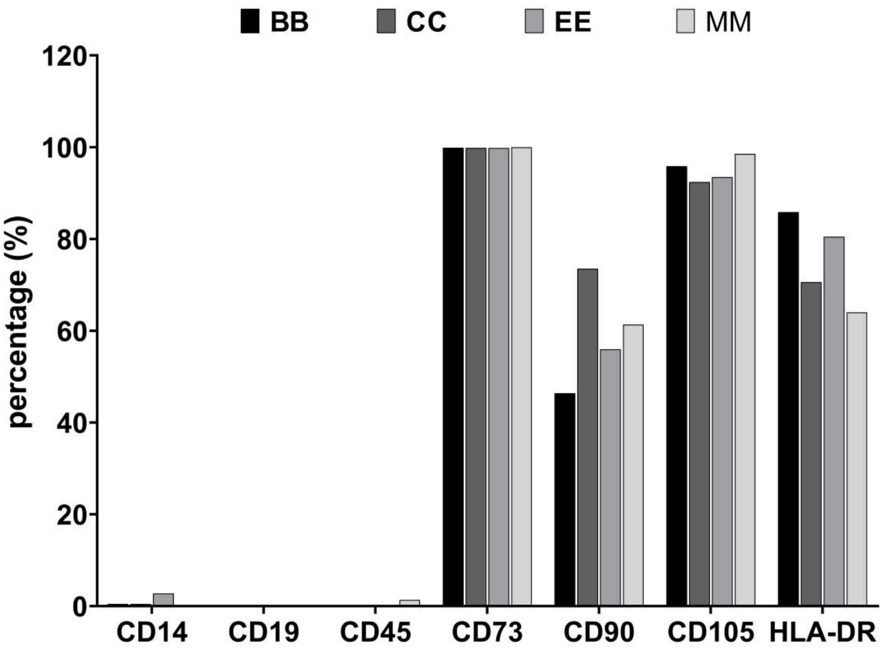

Supplemental Figure S2 (related to Figure 1). Flow cytometric data of four representative MSC populations characterized by osteogenic differentiation in Figure 1 and Supplemental Figure S1.

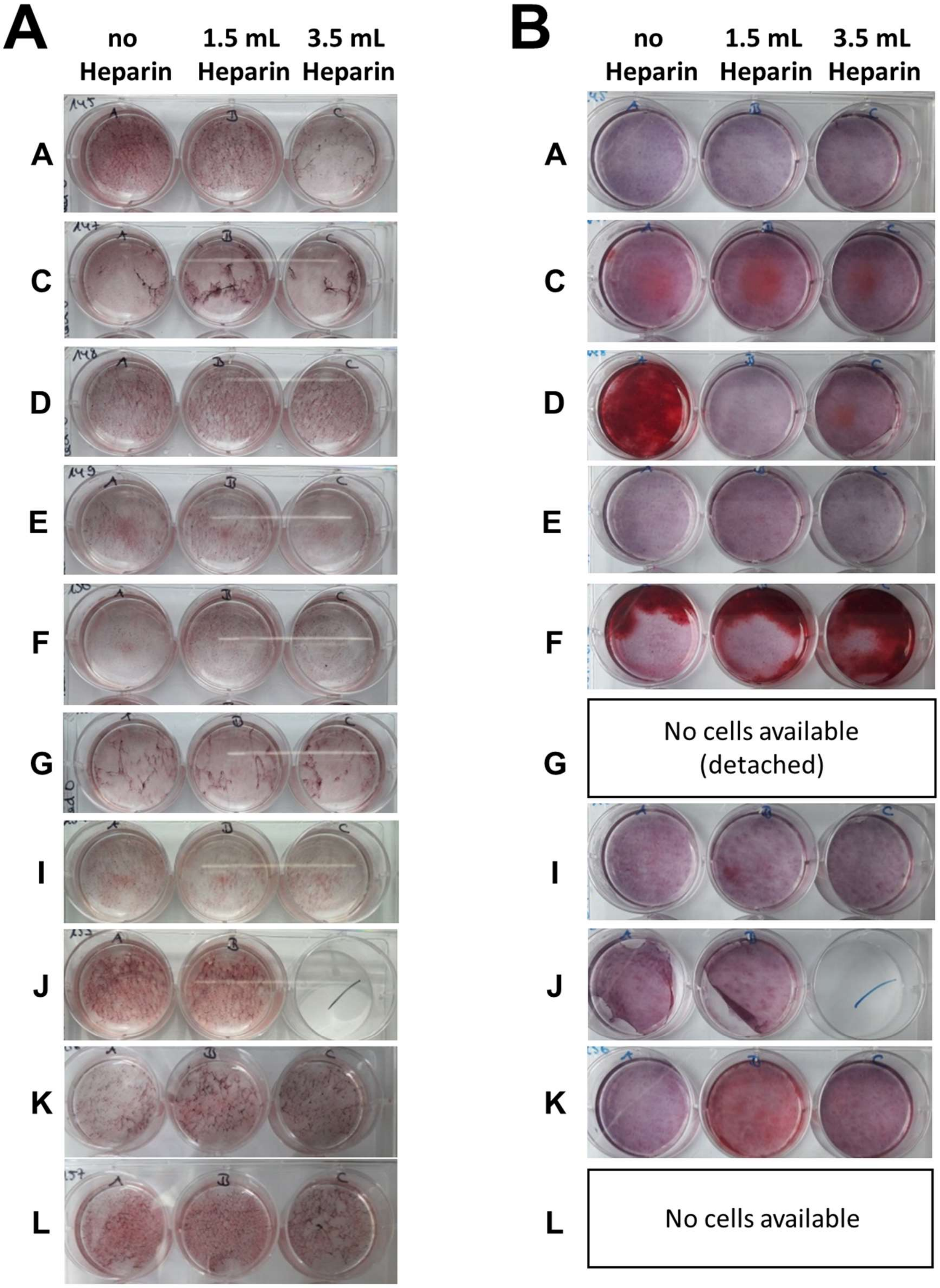

Supplemental Figure S3 (related to Figures 5 and 6). Results of *in vitro* differentiation. **(A)** Photos of Oil Red O-stained cell layers in sixwell-plates after 14 days of adipogenic differentiation. **(B)** Photos of Alizarin Red S-stained cell layers in sixwell-plates after 27 days of osteogenic differentiation.

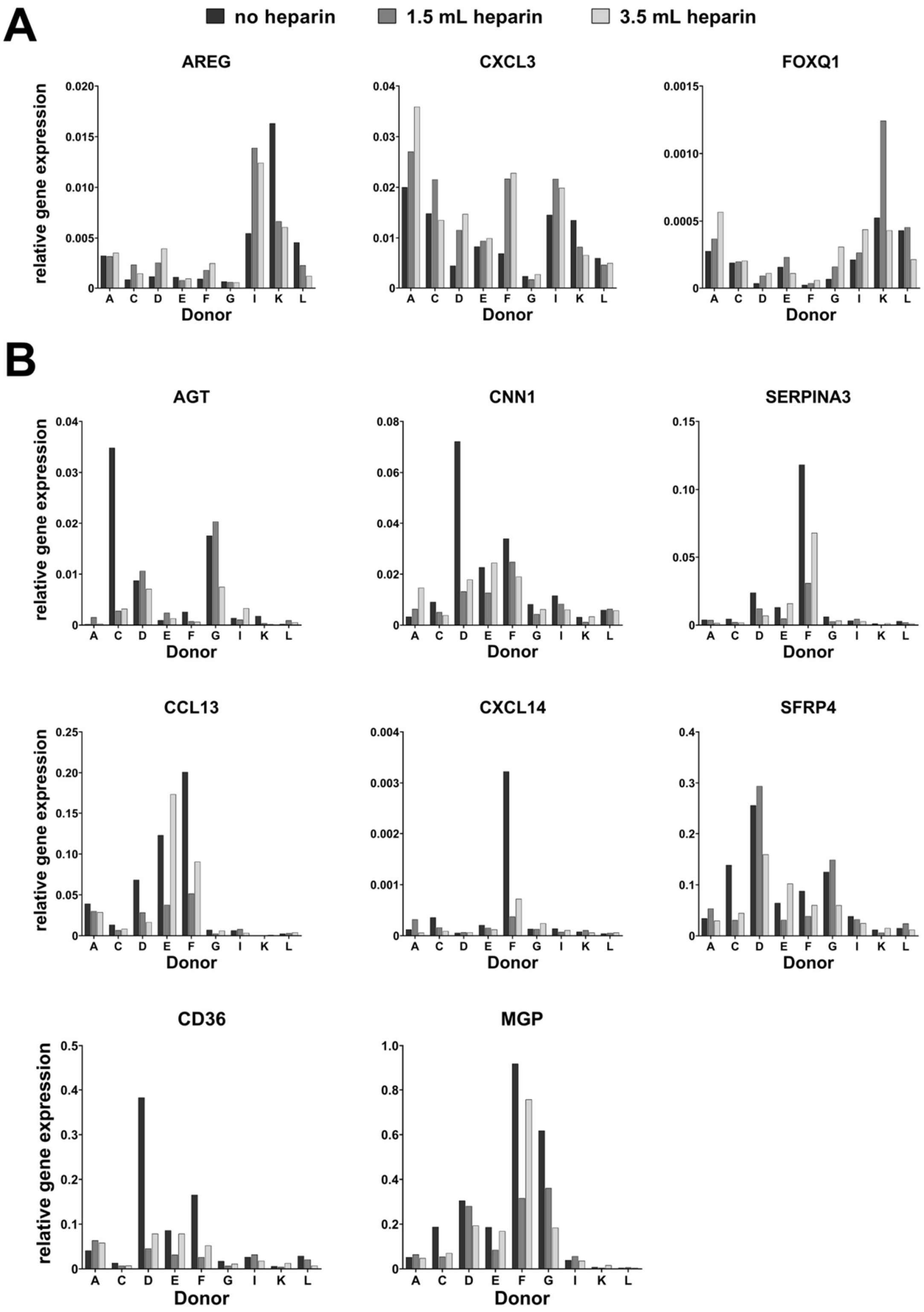

Supplemental Figure S4 (related to Figure 8). qRT-PCR analysis for genes selected from the results of transcriptome analyses. **(A)** Relative gene expressions ($2^{-\Delta Ct}$) of three genes identified to be upregulated by heparin. **(B)** Relative gene expressions ($2^{-\Delta Ct}$) of eight genes identified to be downregulated.
